# Pregnancy outcomes of women exposed to laninamivir during pregnancy

**DOI:** 10.1002/pds.3684

**Published:** 2014-07-29

**Authors:** Hisanori Minakami, Takahiko Kubo, Akihito Nakai, Shigeru Saito, Nobuya Unno

**Affiliations:** 1 Department of Obstetrics, Hokkaido University Graduate School of MedicineSapporo, Japan; 2 Department of Obstetrics, National Center for Child Health and DevelopmentTokyo, Japan; 3 Department of Obstetrics and Gynecology, Tama Nagayama Hospital, Nippon Medical SchoolTokyo, Japan; 4 Department of Obstetrics and Gynecology, University of ToyamaToyama, Japan; 5 Department of Obstetrics and Gynecology, Kitasato UniversityKanagawa, Japan

**Keywords:** antiviral drug, influenza, laninamivir, pregnancy, teratogen, pharmacoepidemiology

## Abstract

**Purpose:**

The purpose of this study is to assess pregnancy outcomes of women treated with a novel neuraminidase inhibitor, laninamivir, during pregnancy.

**Methods:**

A retrospective review of pregnancy outcomes of 112 pregnant women who were given laninamivir for treatment of influenza was performed. Possible adverse events, including miscarriages, preterm birth, foetal malformation and any neonatal morbidity requiring treatment, were assessed.

**Results:**

Seventeen, 39, 46 and 10 women were administered a single inhaled dose of 20 or 40 mg of laninamivir at gestational week (GW) 3–11, 12–21, 22–36 and 37 or more, respectively. One (1.8%) of 56 women with laninamivir at GW <22 experienced miscarriage at GW <12. The remaining 111 women gave birth to 111 viable infants but at preterm (GW <37) in nine (8.8%) of 102 women with laninamivir at GW <37. Three (2.7%) of the 111 newborns had malformations: forefoot varus deformity, foot polydactyly and cleft lip in one each born to a mother taking laninamivir at GW 6, 17 and 21, respectively. Five neonates (4.5%) were small for gestational age. Eleven (9.9%), five (4.5%) and no neonates required phototherapy for jaundice, transient respiratory supports for respiratory distress syndrome (*n* = 2) or transient tachypnoea of the newborn (*n* = 3), and glucose administration for hypoglycaemia, respectively.

**Conclusions:**

Although this study included a small number of study women and no control women, the results suggested that maternal exposure to laninamivir did not increase the rate of adverse pregnancy and foetal outcomes.

## INTRODUCTION

Based on data from seasonal influenza and the 2009 H1N1 pandemic, pregnant women are more severely affected with influenza than the general population.^1^ During the 2009 H1N1 pandemic, early treatment of pregnant women with antiviral medications was associated with fewer admissions to an intensive care unit (ICU) and fewer maternal deaths.^1^ These data emphasise the importance of treatment with antiviral drugs such as oseltamivir or zanamivir for pregnant women with suspected or confirmed influenza. However, the development of resistance to such antiviral drugs in circulating influenza viruses is the concern when a limited number of drugs are available.

Another new neuraminidase inhibitor for inhalation, laninamivir, has recently been developed.^2^ This drug has undergone phase 2 trials in several countries, including Australia, Canada, France, Germany, UK and USA (cited on 8 February 2014, available from URL: http://www.clinicaltrials.gov/ct2/show/NCT01793883?term=igloo&rank=1), and has been used for the treatment of influenza since October 2010 in Japan. According to the package insert, this drug works as a long-acting neuraminidase inhibitor, and a single inhaled dose of 40 mg alone is effective for the treatment of influenza. However, its safety during pregnancy has not been studied.

## METHODS AND RESULTS

We asked members of the Japan Society of Obstetrics and Gynecology and Japan Association of Obstetricians and Gynecologists about their experience regarding the prescription of laninamivir for the treatment of influenza in pregnant women twice in April 2012 and April 2013. Of 50 physicians who reported having prescribed the drug during the first study period between 1 October 1 2011 and 31 March 31 2012 and during the second study period between 1 October 2012 and 31 March 2013, 25 physicians working for 25 different independent facilities located over a wide area of Japan consented to providing information of all women in whom laninamivir was prescribed during pregnancy. All of the 25 physicians declared in written form that they listed all of 112 women who were given laninamivir during the study period at their facilities and provided information relevant to this study including any neonatal abnormalities detected during the stay at their obstetrical facilities.

All 112 women were given laninamivir (20 mg in four women and 40 mg in 108 women) for treatment of influenza that was diagnosed definitely or based on clinical symptoms only, and all of them recovered from influenza completely. They took laninamivir at GW 22.7 ± 10.6 (range: 3–41) and gave birth at GW 38.7 ± 1.4 (range: 34–41) after excluding one who took laninamivir at GW 5 before confirmation of foetal cardiac activity and experienced spontaneous abortion at GW <12 (Table 1). Preterm births at GW <37 occurred in nine women. Two of the nine with preterm birth gave birth several days after taking laninamivir at GW 34 and 36 while suffering from influenza. With the exception of one pregnancy that ended in miscarriage, all women had viable infants weighing 3058 ± 346 (range: 2190–3796) g. Four other adverse episodes within several days after taking laninamivir were recorded in three mothers: vaginal bleeding from the erosive uterine cervix occurred 2 days after taking laninamivir in one, toothache as a result of a decayed tooth occurred 3 days after taking laninamivir in one, and bacterial pneumonitis and premature labour occurred 2 and 4 days after taking laninamivir in one, respectively. All of the three women gave birth to full-term healthy infants.

**Table 1:** Overall characteristics of 112 women

Age (year)	30.4 ± 4.8 (18–40)
<20	1 (0.9% [0.0–4.9])
≥35	29 (25.9% [18.1–35.0])
Nulliparity	42 (37.5% [28.5–47.2])
Alcohol ingestion	0 (0.0% [0.0–3.3])
Smoking habit	5 (4.5% [1.5–10.1])
Caesarean delivery	25 (22.3% [15.0–31.2])
Gestational week	
Laninamivir*	22.7 ± 10.6 (3–41)
At delivery	38.7 ± 1.4 (34–41)†
–21	1 (0.9% [0.0–4.9])
22–33	0 (0.0% [0.0–3.3])
34–36	9 (8.0% [3.7–14.7])
37–	102 (91.1% [84.2–95.6])
Infant birth weight (g)	3058 ± 346 (2190–3796)†
<2500	3 (2.7% [0.6–7.7]†)
Small for gestational age‡	5 (4.5% [1.5–10.2]†)
Respiratory distress syndrome	2 (1.8% [0.2–6.4]†)
TTN§	3 (2.7% [0.6–7.7]†)
Hyperbilirubinaema¶	11 (9.9% [5.1–17.0]†)
Hypoglycaemia¶	0 (0.0% [0.0–3.3]†)
Cacridovascular malformation¶	0 (0.0% [0.0–3.3]†)
Malformed infant	3 (2.7% [0.6–7.7]†)

Data are presented as means ± standard deviation (range, minimum–maximum) and proportion (% [95% confidence interval]).

Not all of the infants were screened for cardiac structural anomalies with echocardiography in this study.

* With laninamivir.

† Among 111 neonates after excluding one pregnancy that ended in miscarriage.

‡ Birth weight less than 10th percentile for Japanese infants for each gestational week.^3^

§ Transient tachypnoea of the newborn.

¶ Those requiring treatment.

Eight, 9, 39, 46, and 10 women took laninamivir at GW 3–7, 8–11, 12–21, 22–36 and 37 or later, respectively (Table 2). A total of 26 abnormalities other than low birth weight <2500 g were detected in 22 (20%) of the 111 neonates during 6.6 ± 1.8 (range: 1–12 days) day stay in the obstetrical facility: three malformed infants (bilateral forefoot varus deformity, left foot polydactyly and cleft lip) born to three women exposed to laninamivir at GW 6, 17 and 21, respectively, (case with forefoot varus deformity born at GW 40 weighing 2630 g also had small for gestational age [SGA]^3^ and transient tachypnoea of the newborn [TTN], case with left foot polydactyly was born at GW 40 weighing 2755 g, and case with cleft lip was born at GW 41 weighing 3000 g), SGA in five (one complicated with impetiginous eczema), hyperbilirubinaemia requiring phototherapy in 11 (one complicated with respiratory distress syndrome [RDS]), RDS or TTN in five, and others in two (congenital bacterial infection and impetiginous eczema in each one). RDS occurred in two neonates born at GW 34 and 35. TTN occurred in three neonates born at GW 36, 40, and 40. Neither hypoglycaemia nor cardiac malformations were detected in any neonate.

**Table 2:** Events occurring after exposure to laninamivir

	GW at delivery			Neonates				
GW at exposure	≤21	22–36	≥37	Malformed	SGA	HB	RDS/TTN	Others
0–7 (*n* = 8)	1	2	5	1	1	0	0/2	0
8–11 (*n* = 9)	0	1	8	0	0	2	0/0	0
12–21 (*n* = 39)	0	3	36	2	1	5	1/1	1†
22–36 (*n* = 46)	NA	3	43	0	2	4	1/0	1¶
37–(*n* = 10)	NA	NA	10	0	1	0	0/0	0
Overall (*n* = 112)	1	9	102	3	5	11	2/3	2
	(1.8%‡)	(8.8%‡)	(91.0%‡)	(2.7%*)	(4.5%*)	(9.9%*)	(4.5%*)	(1.8%*)
[95%CI]	[0.0–9.6]	[4.1–16.1]	[84.2–95.6]	[0.6–7.7]	[1.5–10.2]	[5.1–17.0]	[1.5–10.2]	[0.2–6.4]

CI confidence interval, GW at exposure gestational week at exposure to laninamivir, HB hyperbililubinaemia requiring phototherapy, SGA small for gestational age, NA not applicable, RDS respiratory distress syndrome, TTN transient tachypnoea of newborn.

* The number of infants was 111 after excluding one pregnancy that ended in miscarriage.

† Congenital bacterial infection.

¶ Impetiginous eczema.

‡ percentage among women who took laninamivir before events.

## DISCUSSION

No increased risks of miscarriage, preterm births, foetal malformation or neonatal adverse events, such as SGA, jaundice requiring phototherapy or respiratory dysfunction including RDS and TTN appeared to occur in mothers who were exposed to laninamivir during their pregnancies compared with those of general population.

Three infants (2.7%) were affected by malformations in this study, that is, forefoot varus deformity, foot polydactyly and cleft lip. These malformations are uncommon, with rates of 0.1% for congenital clubfoot occurs,^4^ 0.1% for congenital polydactyly including hand and foot polydactyly as a single malformation^5^ and 0.04–0.2% for congenial cleft lip.^6^ However, the rate of major congenital abnormalities other than neural tube defects and genetic syndromes diagnosed up to the eighth month of life is 2.8%.^7^ Furthermore, two infants with foot polydactyly and cleft lip were born to women who were exposed to laninamivir at GW 17 and 21, respectively, suggesting that these anomalies were not associated with the drug. Although this study was not conclusive regarding the teratogenicity of laninamivir as a result of the small number of women treated with laninamivir during the ‘period of organogenesis’, the results presented here may provide some reassurance to women who are exposed to laninamivir during pregnancy.

Early spontaneous abortion at GW <12 occurred in one (5.9%) of the 17 women who were exposed to laninamivir at GW <12, which was not greater than the general prevalence rate of 13.5%.^8^ The preterm birth rate of 8.8% (9/102) among women exposed to laninamivir before reaching GW 37 in this study was somewhat higher than that of 5.8% in the general Japanese population.^9^ However, two of the nine women with preterm delivery gave birth several days after taking laninamivir while suffering from influenza. Severe influenza-related complications are risk factors for preterm birth,^1^,^9^ with a 2.5-fold higher risk of preterm birth in patients who required hospitalization for treatment of influenza than in the general population.^9^ Thus, laninamivir was considered not to have been responsible for preterm birth. No increased risk of preterm birth was observed in women exposed to other neuraminidase inhibitors, such as oseltamivir and zanamivir.^10^,^11^

Hyperbilirubinaemia requiring phototherapy was seen in 9.9% (11/111) of infants exposed to laninamivir *in utero* in this study. Similar prevalence rates of hyperbilirubinaemia were reported irrespective of exposure to oseltamivir; hyperbilirubinaemia in 9.0% (12/133) and 8.0% (6344/79549) of infants with and without exposure to oseltamivir *in utero*, respectively.^12^ Although a significantly higher risk of hypoglycaemia was reported among infants exposed to neuraminidase inhibitor in one study (4.7% [4/86] vs 1.2% [10/860]; adjusted odds ratio and [95% confidence interval], 5.26 [1.47–18.82]),^13^ no infants suffered from hypoglycaemia in this study. No increased risk of SGA was reported among infants exposed to neuraminidase inhibitors,^10^,^12^,^13^ as was confirmed in this study; the prevalence of SGA was 4.5% (5/111) in this study, while the expected number of SGA neonates was 10% (around 11 of the 111 neonates). RDS occurring in two neonates born at GW 34 and 35 may have been because of prematurity. Although TTN is also likely to occur in preterm infants, it occurs in 1.1% of full-term infants^14^ and up to 3.5% of all infants with Caesarean delivery at or after GW 35.^15^ Two of the three with TTN were full-term infants, accounting for 2.0% of 102 full-term infants, not markedly exceeding the expected risk of TTN.

There were possible drawbacks such as recall and selection biases in this study. As only 50% of physicians participated in this study, physicians with a particularly good, or a particularly adverse, experience with laninamivir may have been more motivated to participate. No control women were included in this study. However, results of this study suggested that no increased risks of adverse events occurred after taking laninamivir compared with that of general population, consistent with results of previous studies on other neuraminidase inhibitors, such as oseltamivir and zanamivir.

## CONFLICT OF INTEREST

All of the authors are medical advisors of this study and were granted the advisor fee from Daiichi Sankyo Co., Ltd. This study was sponsored by Daiichi Sankyo Co. Ltd., and data collection was performed by Daiichi Sankyo Co. Ltd.

**KEY POINTS**

- Laninamivir is a novel neuraminidase inhibitor.
- Pregnancy outcomes were assessed in 112 women treated with laninamivir during pregnancy.
- Maternal exposure to laninamivir did not appear to increase the risk of adverse outcomes in this small study.

## ETHICS STATEMENT

This retrospective cohort study was conducted to determine whether laninamivir used in pregnancy increased the risk of adverse pregnancy outcomes in compliance with the Good Post-marketing Study Practice by the Japan Ministry of Health, Labour, and Welfare Ordinance number 171 and after receiving approval from the Institutional Review Board of Hokkaido University Hospital.
